# Economic Impact of NMMO Pretreatment on Ethanol and Biogas Production from Pinewood

**DOI:** 10.1155/2014/320254

**Published:** 2014-09-07

**Authors:** Marzieh Shafiei, Keikhosro Karimi, Hamid Zilouei, Mohammad J. Taherzadeh

**Affiliations:** ^1^Department of Chemical Engineering, Isfahan University of Technology, Isfahan 84156-83111, Iran; ^2^Industrial Biotechnology Group, Institute of Biotechnology and Bioengineering, Isfahan University of Technology, Isfahan 84156-83111, Iran; ^3^Swedish Centre for Resource Recovery, University of Borås, 501 90 Borås, Sweden

## Abstract

Processes for ethanol and biogas (scenario 1) and biomethane (scenario 2) production from pinewood improved by N-methylmorpholine-N-oxide (NMMO) pretreatment were developed and simulated by Aspen plus. These processes were compared with two processes using steam explosion instead of NMMO pretreatment ethanol (scenario 3) and biomethane (scenario 4) production, and the economies of all processes were evaluated by Aspen Process Economic Analyzer. Gasoline equivalent prices of the products including 25% value added tax (VAT) and selling and distribution expenses for scenarios 1 to 4 were, respectively, 1.40, 1.20, 1.24, and 1.04 *€*/l, which are lower than gasoline price. The profitability indexes for scenarios 1 to 4 were 1.14, 0.93, 1.16, and 0.96, respectively. Despite the lower manufacturing costs of biomethane, the profitability indexes of these processes were lower than those of the bioethanol processes, because of higher capital requirements. The results showed that taxing rule is an effective parameter on the economy of the biofuels. The gasoline equivalent prices of the biofuels were 15–37% lower than gasoline; however, 37% of the gasoline price contributes to energy and carbon dioxide tax which are not included in the prices of biofuels based on the Swedish taxation rules.

## 1. Introduction

Ethanol and biomethane are two common biofuels which are already available in the market in some countries. Ethanol is mainly produced from sugar and starch based raw materials. However, wide investigations are performed for replacement of these food based raw materials with cheaper and more abundant lignocellulosic materials [1]. Biogas is currently produced in wastewater treatment plants or from various organic wastes such as municipal solid waste, manure, and industrial and agricultural wastes [2]. Biogas from wastes is mainly used in the power plants, and the compressed biomethane is commercially available beside the compressed natural gas (CNG) as a vehicle fuel. Considering the predicted expansion of methane usage in Sweden, there would be an increasing market for biomethane to be used as a vehicle fuel. Moreover, further developments in natural gas grid support more injection of biomethane in the grid [3].

A pretreatment step prior to biofuel production from lignocellulosic materials is essential for improvement of the low yields [1, 5–9]. Pretreatment with N-methyl morpholine-N-oxide (NMMO) is among the novel and efficient methods [5–7]. NMMO is a nontoxic cellulose solvent, does not produce toxic wastes, and can be recycled over 98% [6]. NMMO pretreatment modifies the structure of lignocellulosic materials to obtain higher yields of enzymatic hydrolysis and anaerobic digestion. During pretreatment, NMMO dissolves cellulose which is inside of the cell wall of wood. Afterwards, addition of water regenerates cellulose, and less crystalline and amorphous cellulose precipitates on the biomass surfaces. The hydrolysis of regenerated cellulose is much more convenient than the intact cellulose inside the cell wall. Furthermore, the pretreatment process increases the biomass porosity and consequently accessibility of degrading enzymes or bacteria to the inside of the biomass. Better accessibility to the inside of biomass results in enhanced yields of enzymatic hydrolysis and anaerobic digestion [5]. One of the important features of NMMO pretreatment is that physical removal of lignin and hemicellulose is not necessary to obtain a high cellulose hydrolysis yield. The modifications made by NMMO pretreatment are so efficient that the subsequent enzymatic hydrolysis yield is significantly higher than the yield after most of the other pretreatments, for example, steam explosion.

Another promising pretreatment process is steam explosion. Although it has a lower efficiency compared with NMMO pretreatment, it is a simple method that is well investigated in laboratory and pilot scales and is suggested for industrial scale applications [1].

Biofuel production from lignocellulosic materials is a developing technology and still challenging with technical and economical bottlenecks. Technoeconomic analysis helps to overcome these problems using process simulation tools together with economic analysis [10]. Several technoeconomic analyses were performed for bioethanol production from lignocelluloses [10–12]. The economics of biogas production from lignocellulosic materials were also studied [2, 3, 13]. However, no reference was detected for technoeconomic comparison of NMMO and steam explosion pretreatment for both bioethanol and biogas production from lignocellulosic materials.

Significant improvements in the ethanol and biogas yields from NMMO treated pinewood were observed [5]. In the current study, based on the experimental results, the economics of the processes for bioethanol and biogas productions with NMMO pretreatment were compared with two processes using continuous steam explosion pretreatment for similar products. The processes were simulated and optimized using Aspen plus, and the economics were evaluated with Aspen Process Economic Analyzer (PEA). A sensitivity analysis was also performed to determine the effective parameters.

## 2. Methods

Four scenarios for production of bioethanol and biogas using steam explosion or NMMO pretreatment were developed. The process for each scenario was simulated by Aspen plus, and then the economy was studied by Aspen PEA. The selected raw material was pinewood because of availability of the experimental results [5]; however, other lignocellulosic feedstocks can be used with some minor modifications.

### 2.1. Process Development

This study includes four main scenarios. In the first two scenarios, NMMO pretreatment is used for improvement of ethanol and biogas production (scenario 1) and for only biogas production (scenario 2). The other two scenarios are for the production of the similar products but steam explosion pretreatment is used instead of NMMO pretreatment (scenarios 3 and 4).

#### 2.1.1. Scenario 1: NMMO Pretreatment for Improvement of Ethanol and Biogas Production

**Figure 1:**
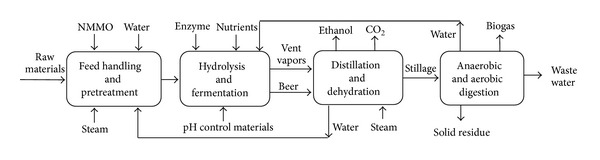
Block flow diagram (BFD) of scenario 1: NMMO pretreatment for production of ethanol and biogas.

**Figure 2:**
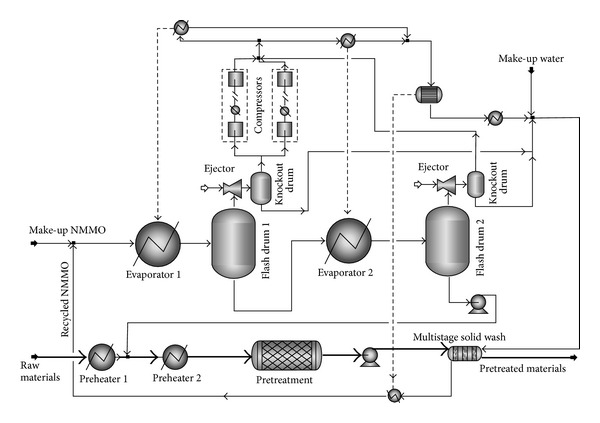
PFD of NMMO pretreatment unit. The optimized mechanical vapor recompression (MVR) system was used for the evaporators (scenarios 1 and 2).

The raw materials are unloaded from trucks to storage area and conveyed for size reduction. All of the scenarios include similar units for the feedstock handling area. In scenario 1 (Figure 1), raw materials are reduced in size and then pretreated with NMMO for 3 hours at 120°C (Figure 2). Then, the materials are regenerated by addition of hot water, washed with water to remove NMMO, and then sent to the biofuel production process. An optimized evaporation unit is used for the recovery and concentration of NMMO (Table 1) [12].

In scenario 1, the wood is washed with water after the pretreatment and the water containing 70% NMMO is sent to evaporation. The evaporation concentrates NMMO to 85%, and it is reused in the pretreatment. A makeup stream for NMMO is considered in the process to supply the amount of NMMO which is not recovered during the washing of the treated wood. Based on the calculations, the NMMO recovery of 99.5% is required to have an economically feasible process. Efficient multistage countercurrent equipment for solid washing is considered to provide this recovery. During the pretreatment, addition of antioxidant agents prevented the oxidation and degradation of NMMO.

Ethanol production includes hydrolysis, nonisothermal simultaneous saccharification and fermentation (NSSF), distillation, and dehydration. After pretreatment, the raw materials are hydrolyzed with Cellic CTec3 enzyme (Novozymes) for 24 hours. It is claimed that this newly developed enzyme has a higher efficiency compared with the previous types of the Cellic enzymes [12, 14]. Because of improvements in the enzyme efficiency, the enzyme is loaded at the rate of 1.8% w/w of cellulose [12]. The hydrolysis temperature is set to 45°C for better stability. Afterwards, the hydrolysate is cooled down to 37°C for SSF fermentation for 24 hours [12]. Four main fermenters and four hydrolysis reactors are designed with volumes of 800 m^3^. For each of the hydrolysis and fermentation reactors, seed fermenters with relative volume ratio of 1 : 10 until volume of 80 l are used for inoculum preparation. All fermenters and auxiliary equipment are made up of stainless steel 304.

**Figure 3:**
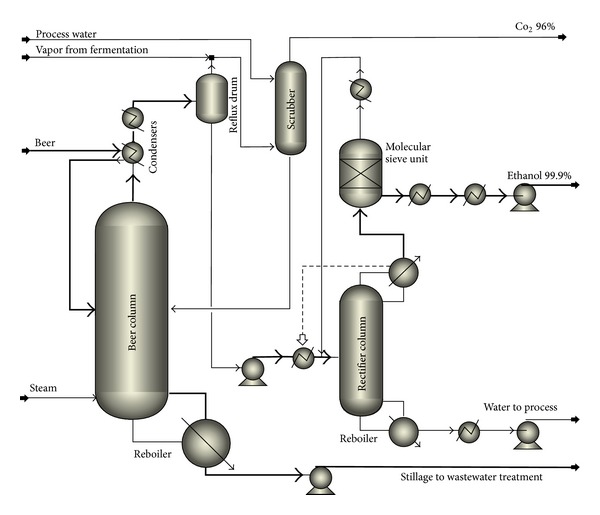
PFD of distillation unit. Beer is processed by a beer column, a rectifier column, and a molecular sieve unit and the product is fuel ethanol (scenarios 1 and 3).

The distillation, dehydration, and wastewater treatment (WWT) units are similar to the systems presented by Shafiei et al. [12] with modifications for the new raw material and lower capacity. The distillation unit system (Figure 3 and Table 2) purifies ethanol to 95.5%. For further purification to 99.9%, a molecular sieve unit is used. The ethanol recovery was assumed to be 96% in the distillation unit. Afterwards, the wastewater from the striper column is filtered for solid removal and then sent to an anaerobic digester of UASB type for biogas production. This system removes 90% of the COD, and the effluent is further purified using aerobic digestion [12]. The biogas produced in this process is not sufficient to have an economically feasible upgrading; therefore, it is sold to a nearby combined heat and power (CHP) plant. An amount of 6% biogas loss was assumed during the storage [15].

#### 2.1.2. Scenario 2: NMMO Pretreatment for Improvement of Biogas Production

**Figure 4:**
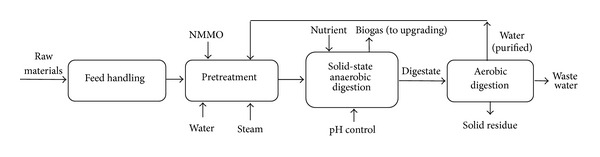
BFD of scenario 2: NMMO pretreatment for production of biomethane.

**Figure 5:**
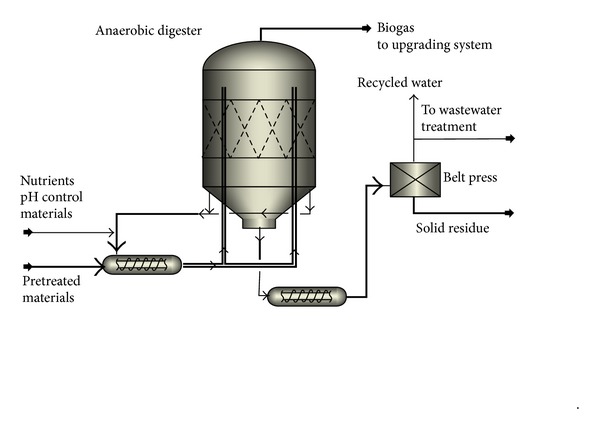
PFD of solid-state biogas production unit (scenarios 2 and 4).

The block flow diagram (BFD) for scenario 2 is presented in Figure 4. Similar to scenario 1, units for feed handling and NMMO pretreatment were assumed for this scenario. The treated materials are then conveyed to the solid-state biogas production unit presented by DRANCO (Germany) [16] (Figure 5). Seven digesters with volume of 3200 m^3^ made of acid resistant coated carbon steel are used in the process. The digesters are vertical cone bottom vessels and are fed using screw pumps. A portion of the outflow is mixed with the pretreated wood and nutrients are sent back to the top of digesters so the overall retention time of materials is 20 days [13, 16]. The digested materials are dewatered to 30% solid content and sold as a byproduct for combustion. Macrofilters and reverse osmosis system are used for water purification, while 80% of the water is recycled to the process [17]. The effluent water is treated using aerobic digestion [12]. The produced biogas is upgraded to 97% with water scrubbing technology with regeneration and then pressurized for further application as fuel. Methane losses are estimated to be 1.5% in the upgrading process and 6% during the storage [15].

#### 2.1.3. Scenario 3: Steam Explosion Pretreatment for Improvement of Ethanol and Biogas Production

**Figure 6:**
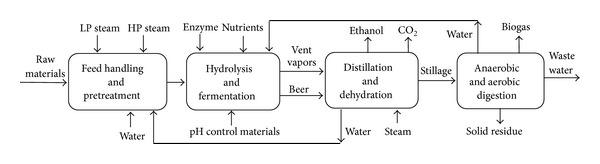
BFD of scenario 3: steam explosion pretreatment for production of ethanol and biogas.

**Figure 7:**
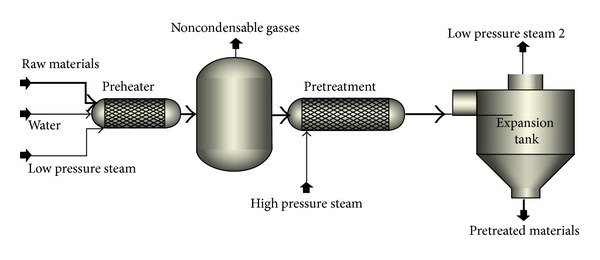
PFD of steam explosion pretreatment unit (scenarios 3 and 4).

The BFD for scenario 3 is presented in Figure 6. The feedstock is handled in an area similar to the previous scenarios. Afterwards, the feedstock is conveyed to the pretreatment area where it is subjected to continuous steam explosion pretreatment (Figure 7). The process design was similar to the process presented by Shafiei et al. [13] with some modifications for the new raw material and lower capacity. Briefly, the system consists of three parallel pretreatment units, each of them has screw conveyors, presteamer, flash vessel, pretreatment reactor, and expansion tank. The treated materials are used for bioethanol production in a process similar to scenario 1. Moreover, the dehydration and wastewater treatment units are similar to scenario 1 [12]. The raw biogas is sold as a byproduct for heat and power generation.

#### 2.1.4. Scenario 4: Steam Explosion Pretreatment for Improvement of Biogas Production

**Figure 8:**
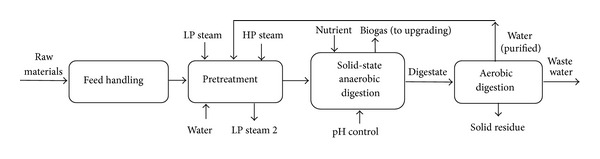
BFD of scenario 4: steam explosion pretreatment for production of biomethane.

In scenario 4 (Figure 8), the feedstock handling and pretreatment area are similar to scenario 3. However, the materials are sent for solid-state biogas production in a process similar to scenario 2. The water from the process is purified using macrofilters and reverse osmosis system and partially recycled to the process. Complete recycling is not possible due to accumulation of some ions and chemicals in the process. The biogas is upgraded and pressurized in a process similar to scenario 2 [17, 18].

### 2.2. Plant Location and Capacity

Sweden was selected for the plant location because of its large biofuel vehicle fleet in Europe [19]. In order to support the economy of the biofuel production plant, it is necessary to locate it nearby a CHP plant. In such a way, a part of capital costs for steam and electricity production is reduced. Several CHP plants are already built in Sweden for production of energy from wood, municipal waste, and forest biomass. Most of the CHP plants in the main cities of Sweden are large enough to support the electricity and steam requirement of the biofuel plant. For instance, each of the CHP plants in Stockholm area produces 800–1700 GW heat and 200–750 GW electricity [20]. Finally, the availability of raw material and transportation costs would affect the final decision for exact selection of the plant location.

Wood is already used in Sweden for energy production. For example, in Brista plant in Stockholm, 350,000 ton per year of wood chips is used [20]. In this study, the plants were designed for utilization of 100,000 ton/year pinewood which have a half of the capacity of previous studies [12]. The wooden raw material required for this plant is around 1% of the total amount of 16 million m^3^ of sawn wood (spruce and pine) produced in Sweden [21].

Biogas, the byproduct of scenarios 1 and 3, can be sold to the CHP plant for combustion. Solid residue, another byproduct of the processes, contains about 30% dry material. Over 70% of the dry material of solid residue is lignin, and other main materials are cellulose, hemicellulose, and biomass. Solid residue may be further used in gasification, pyrolysis, or combustion processes. However, presence of water in the solid residue is one of the major challenges for gasification and pyrolysis [22]. Thus, solid residue is sold to the CHP plant for burning.

### 2.3. Process Simulation and Economic Evaluation

The main equipment of the four processes was simulated by Aspen plus simulation software. Unique features of this software are handling of materials in solid state and broad property data bank, which are beneficial for the best design, simulation, and optimization of the processes [23]. The software does the rigorous calculations for the equipment using a detailed model and determines the mass and energy in all streams of the process. For the physical and thermodynamic properties of the wood, a data bank prepared by NREL (National Renewable Energy Laboratory, USA) [24] was introduced to the software.

Based on the results from simulation, equipment sizing and optimization were performed using Aspen plus and Aspen PEA. Afterwards, the costs were estimated for all major equipment with Aspen PEA. The cost for some units was estimated based on the literature: ethanol dehydration unit [25], steam explosion equipment [25], and biogas upgrading and pressurizing [26]. Basic assumptions for economic evaluation are similar to the previous studies [13] with the following modifications.

- The capacity is reduced to 100,000 ton of dry materials per year.
- Chemical engineering cost index of 2014 was used for the cost estimations.
- The construction periods for scenarios 1 to 4 are 20, 36, 21, and 30 weeks per Aspen PEA suggestion.

The manufacturing costs of ethanol and biomethane were calculated according to the method presented by Peters and Timmerhaus [27]; however, the credit of the byproducts was subtracted from the manufacturing cost.

### 2.4. Sensitivity Analysis

A sensitivity analysis was performed to determine the most effective parameters (among the raw materials and byproducts) in the economy of the process. For the better comparison of four scenarios, the gasoline equivalent prices of the products were calculated using the lower heating values of the fuels which are 36.1 MJ/Nm^3^ for biomethane, 21.2 MJ/l for bioethanol, and 32.0 MJ/l for gasoline.

## 3. Results and Discussion

Based on the experimental results [5], two scenarios for production of bioethanol and biogas using NMMO pretreatment were developed. The economy of these two scenarios was compared with the economy of two similar scenarios with steam explosion pretreatment.

### 3.1. Mass and Energy Balances

Four scenarios for the production of bioethanol and biogas were simulated by Aspen plus (Figures 2, 3, 5, and 7). Based on the simulation results, the required raw materials and utilities as well as product specifications are shown in Table 3. Because of the better efficiency of the NMMO pretreatment, the amount of ethanol and biogas in scenarios 1 and 2 was higher compared to scenarios 3 and 4. Therefore, better hydrolysis and digestion in scenarios 1 and 2 lead to production of less solid residues. After steam explosion, the exhaust steam from the expansion tanks can be returned to the CHP plant to be reused in the process. This steam contains 0.15% volatile furans which must be removed before being reused. Carbon dioxide is produced in all of the processes. The purity of carbon dioxide from bioethanol process is over 99%, and it can be sold as a byproduct; however, in the biomethane scenarios, it contributes to about 50% of the raw biogas and cannot be sold.

### 3.2. Total Project Investment

Total project investments calculated by Aspen PEA for scenarios 1 to 4 were 44.0, 69.7, 40.5, and 65.1 million €, respectively. The required capital for NMMO pretreatment was significantly higher than that for the steam explosion pretreatment (Table 4). Although ethanol production required more operating units, that is, hydrolysis, distillation, and dehydration, the facilities for biogas production were more expensive than the equipment required for ethanol production. The digesters were more expensive since the anaerobic digestion requires longer retention time of the materials (20 days) compared with 48 hours for the hydrolysis and fermentation in the ethanol production (Table 4). Additionally, the capital for the biogas upgrading and pressurizing was more expensive compared with the equipment for distillation and dehydration of ethanol.

### 3.3. Cost Distributions

**Figure 9:**
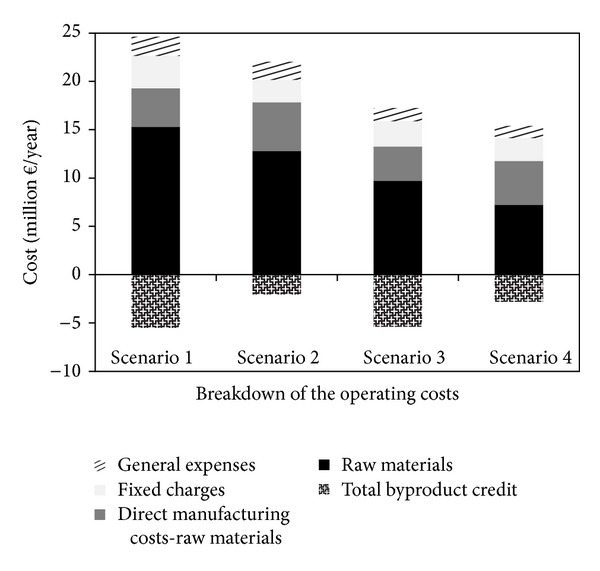
Breakdown of the operating costs for different scenarios.

The breakdown of the operating costs is depicted in Figure 9. The direct manufacturing costs include the costs for raw materials; operating labor and direct supervisory; utilities, maintenance, and repairs; and operating charges. The fixed charges include 30% taxation on the plant income as well as the plant overhead. General expenses include the costs for research and development and financing (10% return rate) and administrative costs. NMMO, lignocellulosic feedstock, and the enzymes are the most costly raw materials. The byproducts of bioethanol plants are biogas, solid residue, and carbon dioxide, while solid residue is the only byproduct of the biogas plants.

### 3.4. Manufacturing Costs and Gasoline Equivalent Prices

In Sweden, taxes are applied on the plant income as well as 25% value added tax on the final price of the products. Additionally, two other taxes are applied on the fossil fuels, but not on the biofuels, which are taxes for energy and carbon dioxide. The amounts of these taxes for gasoline were correspondingly 2.97 and 2.38 SEK/l in 2013. Therefore, tax contributes to 58% of gasoline price [4]. The portion of each of the taxes on the final prices of the biofuels is presented in Table 5. The average price of E85 (fuel ethanol) [28] was 1.14 €/l (9.85 SEK/l converted based on the average Euro price in 2013 [29]). The manufacturing costs were calculated with considering all the parameters presented in Figure 9, including 30% tax on the plant income. The manufacturing costs of ethanol for scenarios 1 and 3, excluding VAT and selling and distribution costs, were calculated to be 0.64 or 0.54 €/l, respectively. The final price for bioethanol including the costs for selling and distribution and the taxes for scenarios 1 and 3 would be 0.93 €/l and 0.83 €/l, respectively. These prices are still lower than the fuel ethanol as well as gasoline (Table 5). The manufacturing costs of the biomethane (97%, pressurized, including plant income tax, VAT, and selling and distribution costs) for scenarios 2 and 4 were calculated to be 1.35 and 1.17 €/Nm^3^ methane, respectively.

The gasoline equivalent prices of the final products, ready for selling at station, are presented in Table 5. The gasoline equivalent prices of all scenarios are lower than the average of gasoline price; however, the safe margin for scenario 1 is lower than other processes. Scenario 4 presents the best product price while scenario 1 shows the highest product price. Despite the better efficiency of the NMMO pretreatment, higher capital and higher raw material expenses of this process have led to higher manufacturing costs for ethanol (scenario 1 compared with scenario 3) and biogas (scenario 2 compared with scenario 4). Another point is that the processes for production of biogas were not as profitable as the ethanol processes, since investment costs for biomethane production are significantly higher than those of the bioethanol process.

The gasoline equivalent expenses of E85 and biomethane 100 are 4% and 16% lower than gasoline, and the prices for the four scenarios are 15–37% less than the fossil fuel. However, only addition of energy and carbon dioxide taxes to the fossil fuels helped the competition of biofuels in the fuel market. Furthermore, there are other bonuses for biofuel vehicles, such as discount on car insurance, free parking spaces, lower annual registration taxes, and exemption from Stockholm congestion tax. Note that the manufacturing cost must be lower than the selling price to earn enough profit.

### 3.5. Sensitivity Analysis

**Figure 10:**
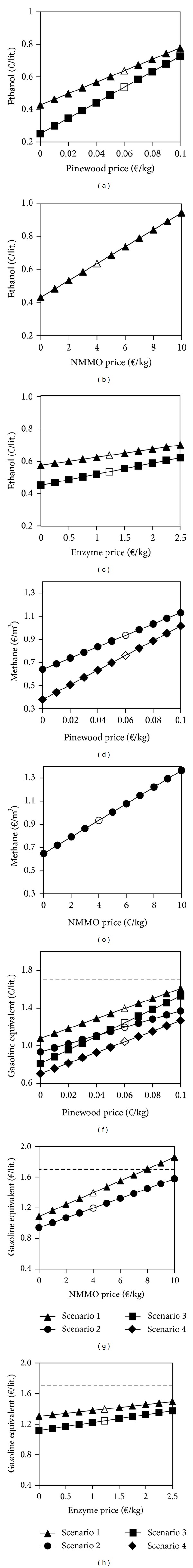
Effects of the price of wood, NMMO, and enzymes on the manufacturing cost of ethanol ((a), (b), and (c)), methane ((d), (e)), and the gasoline equivalent prices ((f), (g), and (h)) for scenarios 1 (▲), 2 (●), 3 (■), and 4 (◆). The dashed line corresponds to average gasoline price in the market. The empty shapes represent the base case values of manufacturing costs. The plant income tax is included in the calculation of the values, but VAT and selling and distribution costs were not added. The gasoline equivalent prices are the prices of ready products and include all expenses (c.f. Table 5).

The effects of price of the most important raw materials on the production cost of ethanol and methane are presented in Figure 10. While other raw materials, for example, nutrients and utilities, did not significantly affect the operating expenses (data not shown), the results of sensitivity analysis indicate that NMMO price had the most significant effect on the manufacturing cost of the products (Figures 10(b), 10(e), and 10(g)) (scenarios 1 and 2). For example, 50% increase in the price of NMMO results in 11% increase in the gasoline equivalent prices. The next two effective parameters are the price of the lignocellulosic feedstock (Figures 10(a), 10(d), and 10(f)) and the enzyme price (Figures 10(c) and 10(h)). Increasing 50% in the wood price has led to 8%, 8%, 13%, and 12% of the gasoline equivalent prices of scenarios 1 to 4, respectively. About 50% increase in the enzyme price for scenarios 1 and 3 has led to 3% and 4% increase in the gasoline equivalent prices, correspondingly. The processes for production of bioethanol had the least safe margin if they are compared with the average petrol price (1.65 €/l) in Sweden market (Figure 10).

**Figure 11:**
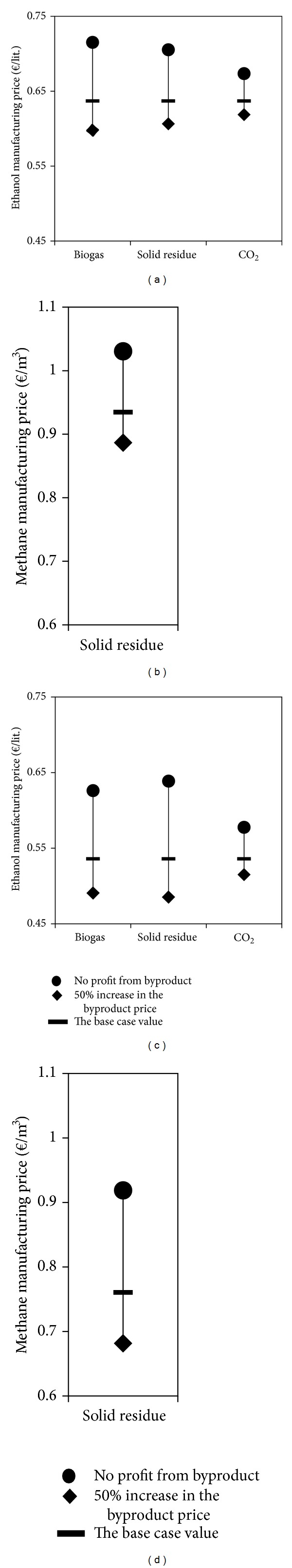
Effect of byproduct price on the manufacturing cost of ethanol and methane for scenarios 1 (a), 2 (b), 3 (c), and 4 (d). The values are prices before addition of VAT and selling and distribution costs.

The effects of byproduct prices on the manufacturing cost of the main products are presented in Figure 11. The data present the comparison of earning no profit from the byproduct or 50% increase in the byproduct price with the base cases. For scenarios 1 and 3, the credit from biogas and solid residue significantly affected the manufacturing costs (Figures 11(a) and 11(c)). For both scenarios 1 and 3, CO_2_ had the least influence on the ethanol price. The price of solid residue was more effective in the manufacturing cost of methane in scenario 4 compared with scenario 2 (Figures 11(b) and 11(d)). The reason was lower efficiency of steam explosion pretreatment compared to NMMO pretreatment which results in lower digestion yield (scenario 4) and production of more solid residues (Table 3).

### 3.6. Profitability of the Processes

Discounted cash flow analysis for each scenario was performed using total capital investment and the annual operating costs. The costs include interests and time value of money. The payback period (payout period) for each scenario was calculated (Table 6) as the minimum length of time to recover the original capital investment. The payback period of the biogas plants was more than that of the bioethanol plant because of the higher capitals requirements. Net rate of return (NRR) shows the profitability of the processes and was calculated by dividing the net present value (NPV) by the present value (PV) of cumulative outflows. The NRR for ethanol production processes was positive while processes for biomethane production had negative NRR (Table 6).

The relative profitability of the processes is presented by profitability index (PI) (Table 6). PI shows the present value of benefits relative to the present value of costs; thus, the PI of a profitable project must be greater than one. The processes for ethanol production (scenarios 1 and 3) were profitable (PI > 1) and the processes for production of biomethane were not profitable (PI < 1).

## 4. Conclusions

Both NMMO and steam explosion led to economically feasible processes for ethanol production (PI > 1); however, none of the biomethane production processes were profitable (PI < 1). Therefore, production of biomethane as the only product from wood may not be economically profitable. However, biogas production from the waste streams of ethanol process considerably helps the economy of the process and reduces the negative environmental impacts. The processes using steam explosion pretreatment were more economically profitable compared to the processes with NMMO pretreatment. Although the pretreatment type significantly affects the yield of final product and consequently the economy of the NMMO process, higher capital as well as more expensive raw materials reduced the overall profitability of the processes with NMMO pretreatment. The technoeconomic study for production of biomethane or ethanol shows that the average gasoline equivalent price of biomethane was 16% lower than that of ethanol and both were 18–39% lower than the taxed gasoline. The energy and carbon dioxide taxes on the gasoline significantly help this competition in favor of the biofuels. Application of cheaper cellulose solvents improves the economy of the process while maintaining high yields of biofuels.

## Figures and Tables

**Table 1:** The process conditions for the equipment of NMMO pretreatment in Figure 2.

Equipment/ conditions	Preheater 1	Preheater 2	Pretreatment	Multistage solid wash	Evaporator 1	Flash drum 1	Evaporator 2	Flash drum 2	Compressor
Input *T* (°C)	20	90	120	120^a^, 45^b^	87.3	79.8	79.8	90	100
Output *T* (°C)	90	120	120	45^a^, 62^b^	79.8	79.8	90	90	170
Input *P* (barg)	0	0	0.5	3	0	−0.78	−0.78	−0.97	0
Output *P* (barg)	0	0.5	0.5	3	−0.78	−0.78	−0.97	−0.97	0.64

^a^The temperature of main streams.

^b^Temperature of washing water.

**Table 2:** The process conditions for equipment of ethanol distillation in Figure 3.

Equipment/ conditions	Beer column	Scrubber column	Rectifier column	Molecular sieve unit
Top input *T* (°C)	50^a^, 40^b^	25	—	—
Bottom input *T* (°C)	110	39	90	106
Top output *T* (°C)	60	39	106	103
Bottom output *T* (°C)	67.6	40	132	103
Top input *P* (barg)	0.5^a^, 0.5^b^	1	—	—
Bottom input *P* (barg)	0.43	0	3	1.9
Top output *P* (barg)	−0.81	−0.1	1.9	0.8
Bottom output *P* (barg)	−0.61	0	2.2	0.8
Number of trays	30	10	35	Packed

^a^The temperature of feed stream.

^b^The temperature of stream from scrubber.

**Table 3:** The amount of raw materials/products and utilities used/produced in each scenario.

	Scenario 1	Scenario 2	Scenario 3	Scenario 4	Price (€/kg)
Raw materials (tpy)^1^					
Pinewood (wet)	105,263	105,263	105,263	105,263	0.06
Nutrients	1100	200	1100	200	0.6
pH control	200	1220	200	1220	0.24/0.15^2^
Enzymes	1,512		1,512		1.226
NMMO	1,536	1,536			4

Products (tpy)^3^					
Methane (m^3^/y)		21,387,468		16,538,970	1.15^4^
Biogas (m^3^/y)	5,952,956		5,217,778		0.75^5^
Solid residue (lignin)	51,317	51,248	56,884	59,112	0.04
LP steam 2^6^			61,912	61,920	0.003
Ethanol (m^3^/y)	30,015		22,132		0.85^7^
CO_2_	21,921		18,480		0.05
Sludge from WWT	250	3,879	232	3,612	0.04

Utilities (tpy)^3^					
Process water	166,324	123,815	121,424	62,264	0.0001
LP steam^6^			30,500	30,500	0.004
HP steam^8^	32,324	3,154	89,171	60,000	0.008
Electricity (Mwh)	17,964	18,076	14,086	14,379	30

^1^tpy: ton per year

^2^The main material for controlling pH in fermentation is NaOH solution (0.24 €/kg). In anaerobic digestion sodium carbonate (0.15 €/kg) is mainly added for maintaining the buffering capacity.

^3^tpy: ton per year, unless stated.

^4^The biomethane is sold at price of 1.15 €/m^3^, which excludes VAT and selling and distribution costs.

^5^The biogas is sold at price of 0.75 €/m^3^, which excludes VAT and selling and distribution costs.

^6^LP steam: low pressure steam.

^7^The price unit is 0.85 €/lit of bioethanol (99.9%). The price excludes VAT and selling and distribution costs.

^8^HP steam: high pressure steam.

**Table 4:** Total project investment and its breakdown for the scenarios.

Scenario	1	2	3	4

Pretreatment	NMMO	NMMO	Steam explosion	Steam explosion

Product	Ethanol/biogas	Biomethane	Ethanol/biogas	Biomethane

Investment cost (million €)				
Feed handling	5.6	5.0	5.6	5.0
Pretreatment	10.2	10.0	6.4	6.4
Hydrolysis and fermentation	9.0	—	10.1	—
Distillation and dehydration	7.8	—	7.7	—
Biogas production	—	21.9	—	21.7
Biogas upgrading/compression	—	21.8	—	21.5
Water treatment	2.3	1.3	2.3	1.3
Utility	4.5	2.6	4.4	2.5
Storage	1.5	3.6	1.3	3.4
Working capital	3.1	3.5	2.7	3.3

Total project investment	44.0	69.7	40.5	65.1

**Table 5:** The manufacturing cost of biofuels and the tax portion of the final prices.

Cost (€/L) or (€/m^3^)	Product cost	30% tax on plant income	Energy tax	Carbon dioxide tax	25% VAT^1^	Final Price	Final price (gasoline equivalent)
Gasoline^2^	0.70	—	0.34	0.28	0.33	1.65	1.65
E85^3^	0.82	—	0.05	0.04	0.23	1.14	1.59
Biomethane 100^4^	1.26	—	—	—	0.31	1.57	1.39

Manufacturing cost^5^							
Bioethanol^6^ (scenario 1)	0.63	0.07	—	—	0.23^7^	0.93	1.40
Biomethane^8^ (scenario 2)	0.97	0.07	—	—	0.31^9^	1.35	1.20
Bioethanol^6^ (scenario 3)	0.50	0.10	—	—	0.23^7^	0.83	1.24
Biomethane^8^ (scenario 4)	0.77	0.09	—	—	0.31^9^	1.17	1.04

^1^VAT is calculated as 25% of the product prices of biomethane which were 10.9 SEK/m^3^ (1.26 €/Nm^3^) and ethanol which were 7.9 SEK/L (0.82 + 0.05 + 0.04 = 0.91 €/L). Therefore, VAT for biomethane = 1.26 ∗ 0.25 = 0.31 €/Nm^3^ and VAT for E85 = 0.91 ∗ 0.25 = 0.23 €/L.

^2^Average of gasoline (95% octane) in 2013 [4]. The gasoline includes 5% bioethanol.

^3^E85 is a blend of bioethanol and 15% gasoline. During winter time, the portion for gasoline increases to 25%. The portion of fossil fuel in E85 includes energy and CO_2_ tax.

^4^Biomethane 100 contains 100% methane from biological sources and is sold in Sweden along with CNG.

^5^Manufacturing cost includes selling and distribution expenses which were 0.06 €/L for ethanol and 0.1 €/Nm^3^ for biomethane.

^6^The plant product is 99.9% bioethanol.

^7^It is assumed that the product will be sold to the market in the same price of E85. Thus, VAT was assumed to be similar to VAT for E85.

^8^The plant product is 97% biomethane.

^9^It is assumed that the product will be sold to the market in the same price of biomethane 100. Thus, VAT was assumed to be similar to VAT for biomethane 100.

**Table 6:** The profitability parameters of the processes.

Scenario	1	2	3	4

Pretreatment	NMMO	NMMO	Steam explosion	Steam explosion

Product	Ethanol/biogas	Biomethane	Ethanol/biogas	Biomethane

Payback period (year)	6.3	8.3	6.2	7.6
Net return rate (NRR) (%)	14.6	−6.3	16.7	−3.0
Profitability index (PI)	1.14	0.93	1.16	0.96
